# From Survival to Productivity Mode: Cytokinins Allow Avoiding the Avoidance Strategy Under Stress Conditions

**DOI:** 10.3389/fpls.2020.00879

**Published:** 2020-07-02

**Authors:** Avishai Avni, Yelena Golan, Natali Shirron, Yeela Shamai, Yaela Golumbic, Yael Danin-Poleg, Shimon Gepstein

**Affiliations:** ^1^Faculty of Biology, Technion – Israel Institute of Technology, Haifa, Israel; ^2^Kinneret Academic College, Sea of Galilee, Israel

**Keywords:** abiotic stress, avoidance, cytokinins, stress tolerance, senescence, transgenic plants

## Abstract

Growth retardation and stress-induced premature plant senescence are accompanied by a severe yield reduction and raise a major agro-economic concern. To improve biomass and yield in agricultural crops under mild stress conditions, the survival must be changed to productivity mode. Our previous successful attempts to delay premature senescence and growth inhibition under abiotic stress conditions by autoregulation of cytokinins (CKs) levels constitute a generic technology toward the development of highly productive plants. Since this technology is based on the induction of CKs synthesis during the age-dependent senescence phase by a senescence-specific promoter (*SARK*), which is not necessarily regulated by abiotic stress conditions, we developed autoregulating transgenic plants expressing the *IPT* gene specifically under abiotic stress conditions. The *Arabidopsis* promoter of the stress-induced metallothionein gene (At*MT*) was isolated, fused to the *IPT* gene and transformed into tobacco plants. The MT:IPT transgenic tobacco plants displayed comparable elevated biomass productivity and maintained growth under drought conditions. To decipher the role and the molecular mechanisms of CKs in reverting the survival transcriptional program to a sustainable plant growth program, we performed gene expression analysis of candidate stress-related genes and found unexpectedly clear downregulation in the CK-overproducing plants. We also investigated kinase activity after applying exogenous CKs to tobacco cell suspensions that were grown in salinity stress. In-gel kinase activity analysis demonstrated CK-dependent deactivation of several stress-related kinases including two of the MAPK components, *SIPK* and *WIPK* and the *NtOSAK*, a member of SnRK2 kinase family, a key component of the ABA signaling cascade. A comprehensive phosphoproteomics analysis of tobacco cells, treated with exogenous CKs under salinity-stress conditions indicated that >50% of the identified phosphoproteins involved in stress responses were dephosphorylated by CKs. We hypothesize that upregulation of CK levels under stress conditions desensitize stress signaling cues through deactivation of kinases that are normally activated under stress conditions. CK-dependent desensitization of environmental stimuli is suggested to attenuate various pathways of the avoidance syndrome including the characteristic growth arrest and the premature senescence while allowing normal growth and metabolic maintenance.

## Introduction

Abiotic stresses, such as drought and salinity are key challenges for plant growth and agricultural productivity, leading to an annual loss of billions of dollars (Pereira, 2016; Zhu, 2016). The evolution of adaptive mechanisms in plants to particular environmental stresses involved activation of stress avoidance strategy that allows plants to escape the potentially damaging effects of these conditions. Under stress conditions, plants activate the stress-avoidance strategy resulting in reduced vegetative growth, leaf shedding, early flowering, accelerated senescence, and loss of biomass/yield (Cramer et al., 2011; Maggio et al., 2018). The stress-avoidance mechanism balances water uptake and water loss. Water uptake is maintained by solute accumulation to lower the cell water potential and by increasing root growth, whereas water loss is restricted by closing stomata, reducing shoot growth, early flowering, and accelerating leaf senescence (Maggio et al., 2018). As the response of plants to water deficit limits biomass/yield, the development of crop varieties with near-normal growth under moderate water stress is critical (Debbarma et al., 2019). Although many breeding programs and genetic engineering technologies have been applied during the last decade, only a few were deemed successful in overcoming this response as it is polygenic and redundantly programmed (Maggio et al., 2018). Survival mode, as reflected in the stress-avoidance response, is often needed in natural environments, but not in most agricultural environments where stresses are less intense and do not persist long enough to threaten survival.

Salinity and drought stresses affect most aspects of plant biology. The pathways associated with the stress-avoidance response include those associated with osmotic and ionic homeostasis, detoxification response, and growth regulation. Osmotic stress activates several protein kinases including mitogen-activated protein kinases (MAPKs), which mediate osmotic homeostasis and/or detoxification responses. In tobacco, the two most investigated stress-related MAPKs are SIPK (48 kD) and WIPK (44 kD) (Seo et al., 1995; Zhang and Klessig, 1997; Romeis et al., 1999; Zhang et al., 2000). Abscisic acid (ABA) biosynthesis is regulated by osmotic stress at multiple steps. Both ABA-dependent and -independent osmotic stress signaling modify constitutively expressed transcription factors (TFs), leading to the expression of early response transcriptional activators, which then activate downstream stress tolerance effector genes (Wu et al., 2007; Yoshida et al., 2014; Du et al., 2018). Osmotic stress induces ABA accumulation by activating its synthesis as well as by inhibiting its degradation (Dong et al., 2015). ABA regulates inhibition of germination, seed dormancy and dehydration, stomata closure, roots elongation during drought stress, and stress-related gene regulation (Hoth et al., 2002; Seki et al., 2002; Xiong et al., 2006; Finkelstein et al., 2008; Cutler et al., 2010). In addition, ABA induces senescence as part of plant development or as a stress-induced response (Gepstein and Thimann, 1980). Abiotic stress-induced responses are regulated by activation of the expression of many genes via ABA-responsive elements (ABREs) in their promoter regions. ABA triggers cascaded activation of SnRK2 that positively controls the AREB/ABF TFs and the S-type anion channel SLAC1 (Fujita et al., 2013; Zhang et al., 2018). The activated anion channels release anions, which are accompanied by the activated K^+^ efflux channels releasing K^+^. As both anion and cation are released from the guard cell turgor is reduced leading to stomatal closure (Roelfsema et al., 2012; Misra et al., 2019). The Ca^2+^ signaling pathway is also involved in the regulation of stomatal aperture through Ca^2+^-dependent protein kinases and CBL (calcineurin B-like protein)-interacting protein kinases. Both kinase systems modulate SLAC1 (Edel and Kudla, 2016).

Drought-responsive genes include effector genes encoding chaperones, enzymes, and ion/water channels, as well as regulatory genes encoding TFs. Several groups of TFs, such as AREB, DREB, NAC, MYB, bZIP, and WRKY, respond to drought stress and act in an ABA-dependent or -independent manner (Joshi et al., 2016). The ABA core signaling pathway largely relies on the activation of SnRK2 kinases to mediate several rapid responses, including gene regulation, stomatal closure, and plant growth modulation. Another kinase, NtOSAK (42 kD), a member of SnRK2 kinase family, is also activated by osmotic stress and ABA in tobacco plants (Kelner et al., 2004; Wawer et al., 2010; Kulik et al., 2011, 2012). Cytokinins (CKs) have a positive role in plant growth and development, but their biosynthesis diminishes during senescence and under water-deficit stress (Chernyad’ev, 2005; Ha et al., 2012). During senescence, applying exogenous CK delays the senescence syndrome (Richmond and Lang, 1957). Since premature senescence negatively impacts yield/biomass, different approaches were used in an attempt to modify the kinetic/severity of the senescence syndrome, especially under stress conditions (Ha et al., 2012; Gepstein and Glick, 2013). Manipulating endogenous CK levels can effectively delay senescence (Gan and Amasino, 1995; Peleg and Blumwald, 2011; Ha et al., 2012; Guo and Gan, 2014). Expression of isopentenyltransferase (*IPT*), the key gene in CK biosynthesis, under different promoters affected plant development, especially on the last phase of the senescence syndrome. Constitutive or inducible expression of the *IPT* gene is associated with arrested senescence phenotype, as well as with loss of apical dominance and altered root growth (Smart et al., 1991; Gan and Amasino, 1995; Peleg and Blumwald, 2011). Inhibition of leaf senescence by autoregulated production of CKs has since been applied to different plants including lettuce, petunia, tobacco, maize, and ryegrass (Jordi et al., 2000; McCabe et al., 2001; Chang et al., 2003; Li et al., 2004; Robson et al., 2004; Peleg and Blumwald, 2011; Golan et al., 2016).

We have previously shown that in transgenic tobacco plants expressing the *IPT* gene under the senescence gene promoter from *Phaseolus vulgaris*, *SARK* (Hajouj et al., 2000), CK is maintained at high levels under water-deficit stress, leading to better survival and increased productivity (Hajouj et al., 2000; Rivero et al., 2007). The molecular mechanisms underlying this have not been fully characterized, although comparative gene-cluster analysis performed in our laboratory suggest that CKs prevent the transcriptional reprograming of known molecular processes associated with stress-tolerance responses (Golan et al., 2016). CKs regulate developmental processes as well as responses to environmental stresses via a complex network of CK signaling (Ha et al., 2012). The receptor gene, *AHK3* is known to partially mediate delayed senescence by phosphorylation of type B *ARR* (Kim et al., 2006). *CRF6* plants begin to undergo monocarpic senescence sooner than WT plants (Rashotte et al., 2006; Zwack and Rashotte, 2013). *CRF6* is induced by CKs and abiotic stresses suggesting possible interaction between *CRF6* and *ARR2* (Cutcliffe et al., 2011). By an unknown mechanism, stress signals are perceived and transmitted via the His-Asp phosphorelay pathway triggering CK-responsive genes (Ha et al., 2012). Multiple mutually interconnected hormonal signaling cascades act as essential endogenous translators of these exogenous signals in the adaptive responses of plants (Verma et al., 2016). Since CKs and ABA are associated with antagonistic inputs in the context of abiotic stress responses, we considered the possibility of interconnection between the two hormonal signaling cascades. Our technology of improving drought tolerance by *IPT* gene driven by *SARK* promoter has been implemented in various crops, including rice, peanuts, creeping-bentgrass, cassava, and tobacco (Rivero et al., 2007; Peleg et al., 2011; Qin et al., 2011; Merewitz et al., 2012).

It is assumed that the main function of leaf senescence is to recycle cellular material accumulated during leaf growth and maturation into exportable nutrients to supply developing organs such as fruits (Avila-Ospina et al., 2014; Maillard et al., 2015). Thus, leaf senescence, due to its role in nutrient management, is essential for plant productivity (Gregersen et al., 2013). Although the transgenic plants (SARK-IPT) display delayed age-dependent senescence and possess an obvious productivity advantage (Rivero et al., 2007), they may also suffer a delay in the optimal harvest timing and a progressive asynchronous fruit ripening and seed set (Lukac et al., 2012). Notably, the timing of triggering senescence is critical for remobilizing mineral nutrients and carbohydrates and may affect fruit quality. The onset of senescence and the rate of its progression also determine the quality of the yield. If the timing is late or the rate is too slow, grains do not dry-down (ripen) completely before harvest, resulting in moisture and nutrients in the harvested material, and consequently, in post-harvest microbial spoilage (Robson et al., 2012).

Since age-dependent senescence is critical for synchronous crop harvest and delaying the normal senescence (by CKs) may interfere with the optimal harvest timing, we decided to focus on delaying the premature stress-induced senescence and allowing normal senescence and proper harvest. Herein, we describe the development of transgenic tobacco plants carrying the *IPT* gene under a stress-specific promoter of the *Arabidopsis* metallothionein (*AtMT*) gene. The rational for choosing the promoter of the MT gene stems from the known functions related to various abiotic stresses of MT genes. Moreover, our previous study which focused on the expression pattern of various senescence-associated genes identified three MT related genes (Gepstein et al., 2003). The *AtMT2* promoter was selected since it represents a general abiotic stress promoter and over-expression of MTs in various model systems like *Arabidopsis*, tobacco, yeast and *E. coli* established its functional role in homeostasis and tolerance to heavy metal ions, high salinity, drought, low temperature, heavy metal ions, ABA, and ethylene. The *MT2a* overexpressing transgenic *Arabidopsis* seedlings had longer roots, larger leaves, and higher biomass accumulation, compared to WT plants under drought, salinity and oxidative stress conditions plants (Patankar et al., 2019). *SbMT-2* gene from a halophyte confers abiotic stress tolerance and modulates reactive oxygen species (ROS) scavenging in transgenic tobacco (Chaturvedi et al., 2014). Since the accumulated literature points to the involvement of the *MT2a* gene in stress-induced senescence phenomenon, we employed the *AtMT2a* promoter to induce *IPT* gene expression and to increase CK levels, specifically under abiotic stress.

Our current results combined with our previous findings in *Arabidopsis* (Golan et al., 2016), suggest that CKs cause desensitization of plants to environmental stress cues and allow plants to escape stress symptoms of the avoidance syndrome, as reflected by sustainable growth and productivity.

## Materials and Methods

### Plants

*Nicotiana tabacum* plants ecotype SR-1, were grown on water-soaked peat pellets (Jiffy 7, Kappa Forenade Well) in temperature-controlled growth room at 23°C (± 2°C) under fluorescent lamps (75 μE m^–2^ s^–1^) under long day conditions (18 h light, 8 h dark). The SR-1 plants were used as a genetic background for the development of transgenic plants. WT and transgenic plants were transferred and transplanted into a greenhouse, using 5 l pots and grown for 25 days (1,200 μmol of photons m^–2^ s^–1^, 16 h photoperiod, 23–25°C day/night). The *N. tabacum* BY-2 cell line (derived from *N. tabacum* Bright Yellow 2) was generously donated by Dr. S. Yaron from the Technion, Israel and maintained by weekly dilution (1:50) in fresh liquid Murashige and Skoog (MS) media (Sigma, St. Louis, MO, United States) supplemented with 0.2 g L^–1^ KH_2_PO_4_, 1 mg L^–1^ thiamine, 0.2 mg L^–1^ 2,4-dichlorophenoxyacetic acid (2,4-D), 30 g L^–1^ sucrose, and 0.2 g L^–1^ myoinositol at pH 6.2, as previously described (Shirron and Yaron, 2011).

### Construction of Transgenic Plants Expressing pMetallothionein:IPT

The plasmid pJHA212K (Yoo et al., 2005) was used as a template to generate the expression vector in which the 35S promoter was replaced by the AtMT promoter. The AtMT promotor (nucleotides 1–1300 bp) selected form the *Arabidopsis AtMT2a* gene (AT3G09390), was fused to the *IPT* gene (coding sequence, nucleotides 1301–2100) followed by the *NOS* terminator nucleotides 2101–2334 generating *AtMT* promoter-*IPT-NOS* terminator construct (Supplementary Figure S1, Gepstein, 2013) and transformed into tobacco SR-1 plants as previously described (Rivero et al., 2007; Golan et al., 2016). The transgenic plants were grown under optimal watering conditions (16 h light, 8 h dark). Five lines of the transgenic plants were characterized, and all displayed similar phenotypes including response to abiotic stresses (data not shown). One representative line (mt7) that showed genetic stability in the transgenic fourth generation (t4) plants was selected for detailed characterization of its morphological and developmental behavior under stress conditions.

### Stress Conditions

#### Drought Stress

One-month old tobacco plants were transferred into a greenhouse (25°± 2°C, 18 h light, 8 h dark) for three more months until full maturation, Plants were watered with 500 ml every 2 days and excess water drained immediately. For the drought stress treatment, after three months, of watering, plant watering was withheld for 3 weeks. The plants were photographed and leaf samples were collected into liquid nitrogen for RNA analysis. Afterward, the plants were re-watered for 1 week and photographed again. Experiments were conducted in triplicates as independent experiments.

#### Salinity Stress

Tobacco plants were grown in 250 ml pots for 3 weeks with tap water watering. Plants were subjected to salinity stress by irrigating every other day with 100 ml of 100 mM NaCl solution during the first 10 days, and with 200 mM NaCl for the subsequent 11 days. Following this period, the plants were photographed and samples from mature leaves were collected into liquid nitrogen for RNA analysis. Experiments were conducted in triplicates as independent experiments. To follow the recovery of the plants after salinity stress, plants were watered with tap water for 2 weeks and photographed.

### BY-2 Cells

BY-2 cell suspension was grown for 5 days in a shaker incubator at 25°C in dark as described (Shirron and Yaron, 2011). The suspension was divided into four 250 ml flasks (50 ml suspension in each) and for the CKs treatments, 6-benzyloaminopurine (BAP), a synthetic CK, was added into the suspension solution to a final concentration of 10 μM. After 1 h of pre-treatment incubation, NaCl was added to a final concentration of 50 mM. For the phosphoproteomics analysis, the suspension was collected into 50 ml conical tubes half an hour later, centrifuged at 4,500 rpm for 2 min at 4°C, and frozen immediately until protein extraction was performed. For kinase activity assays, samples were collected at 5, 10, 20, 30, 90, and 120 min following salt addition and treated as indicated above. Both experiments were conducted in triplicates as independent experiments.

### RNA Extraction

RNA isolation from approximately 150 mg of plant material was performed using the SV Total RNA Isolation kit (Promega #Z3100, Madison, Wisconsin), according to manufacturer’s instructions. Following freezing in liquid nitrogen, the samples were grounded with mortar and pestle in 2 ml Eppendorf tubes in the kit suspension solution. RNA was kept at -70°C.

### Real-Time PCR Reaction

Total RNA was extracted from the leave samples followed by removal of contaminating DNA with RNase-free DNase I. Primers for amplifying selected genes (Supplementary Table S1) were designed using Primer Express software (Applied Biosystems, Foster City, CA, United States). Semi-quantitative expression analyses of RNA were performed by RT-PCR in triplicates under identical conditions using 18S rRNA as an internal control. Concentration, integrity, and extent of contamination by rRNA were monitored using the ND-1000 spectrophotometer (Thermo Fisher Scientific, Waltham, MA, United States). cDNA was prepared from the total RNA with qScript cDNA Synthesis Kit (Quantabio, Beverly, MA, United States) as described by the manufacturer. qRT-PCR was performed using Absolute SYBR Green ROX Mix (ABgene, Portsmouth, NH, United States) or Real Time SYBR Green FastMix ROX (Quantabio, Beverly, MA, United States) kits based on detection of SYBR Green binding to dsDNA. The reaction consisted of 5 μl cDNA, 10 μl SYBR Green mix, 1 μl forward primer (5 pmol), 1 μl reverse primer (5 pmol), and 3 μl double-distilled water (DDW). Reaction conditions were 2 min at 50°C, 15 min at 95°C, and 40 cycles of 15 s at 95°C followed by 1 min at 60°C.

### Protein Extraction

Suspensions of BY-2 cells (5 ml) were centrifuged for 2 min at 4500 rpm at 4°C, and the pellet was quickly frozen with liquid nitrogen. The pellets were re-suspended in 1 ml homogenization buffer [100 mM HEPES pH 8.2, 0.05 mM Sodium Deoxycholate Detergent (Thermo Fisher Scientific, Waltham, MA, United States)] and the samples were sonicated in 3 watt (400,000 J) for 15 s × 4 (TPC-40, Telsonic, Switzerland). Then, samples were centrifuged at 4,500 rpm for 2 min at RT and the supernatant was collected. Protein concentration was determined using Bradford protein assay (Bio-Rad, Hercules, CA, United States) with bovine serum albumin (BSA) as a standard.

### In-Gel Kinase Activity Assay

Cell suspension was exposed to BAP, a synthetic CK, for 1 h and were then subjected to salinity stress (50 mM NaCl) for one additional hour. Kinase activity assay was performed as previously described (Wu et al., 2007) using myelin basic protein (MBP) as a substrate which enabled measuring the phosphorylation activity of the selected kinases. In brief, equal amounts of proteins were separated on 10% SDS-polyacrylamid gel embedded with specific kinase substrate (0.5 mg/ml MBP for SIPK and WIPK, or 0.5 mg/ml histone for NtOSAK). After electrophoresis, the gels were washed three times for 30 min in washing buffer at RT. Then, the gel was treated with a re-naturing buffer and re-incubated in a reaction buffer containing 0.5 mM ATP (Sigma #A26209, St. Louis, MO, United States), with the addition of 50 mCi g-32P ATP (PerkinElmer, Waltham, MA, United States). Subsequently, the gels were transferred into stop solution for 5 h. After washing, the gels were dried on 3 mm Whatman paper and auto-radiographed on Fujifilm film. Finally, photos were obtained with Typhoon FLA 7000 (GE Healthcare, Chicago, IL, United States) phosphor imager system.

### Immunoblotting

Western blot analysis was carried out as previously described (Ben-David et al., 1983), with protein ladder 10–180 kD (Thermo Fisher Scientific, Waltham, MA, United States). Kinases were identified using specific antibodies reacting with protein extracts of BY-2 cell suspension (Seo et al., 2007; Wu et al., 2007). *WIPK* and *SIPK* (members of the MAPK family), are components of the ABA-independent pathway, and have been reported to be activated under abiotic stress (Mikolajczyk et al., 2000; Zhang et al., 2000; Seo et al., 2007). Antibodies against NtOSAK were generously donated by Prof. Grazyna Dobrowolska (Institute of Biochemistry and Biophysics, Polish Academy of Science, Warsaw, Poland), and antibodies against WIPK and SIPK were generously donated by Prof. Shigemi Seo (National Institute of Agrobiological Sciences, Tsukuba, Japan).

### Phosphoproteomics

For phosphoproteomics analysis, proteins were isolated and sent to the Technion’s Smoler protein center. The proteins were digested by trypsin and phosphopeptide enrichment was performed using TiO_2_. The phosphopeptides were analyzed by LC-MS/MS on Q-exactive mass spectrometer (Thermo Fisher Scientific, Waltham, MA, United States). Data were analyzed with maxQuant 1.4.1.2 against Niben.genome.v0.4.4.proteins.wdesc database of *Nicotiana benthamiana* (Bombarely et al., 2012). Quantitative analysis was performed using Perseus software (Tyanova et al., 2016). Phosphopeptides with *p* < 0.05 were analyzed and their function was identified based on their gi number (Sequence Identifier) using NCBI-nr database.

### Proline Quantification

Leaf samples (0.1 g) were frozen in liquid nitrogen and grinded. Thereafter, 1 ml of 3% sulfosalicylic acid was added, samples were centrifuged for 5 min at 14,000 rpm, supernatant was collected, and 0.25 ml from the supernatant were added to 2.75 ml of 3% sulfosalicylic acid, 3 ml acetic acid (glacial), and 3 ml 2.5% ninhydrin solution, mixed gently, and boiled at 100°C for 1 h. After cooling, 3 ml toluene was added to each sample. The samples were vortexed and incubated at RT overnight. The following day, the absorbance of 1 ml from the upper phase was read in spectrophotometer at 520 nm with toluene as reference. For quantification, a standard curve of L-proline (Sigma #P0380, St. Louis, MO, United States) was used.

### Na^+^ and K^+^ Quantification by Optical Emission Spectrometry (ICP) Analysis

Mature, fully expanded, tobacco leaves were dried at 80°C for 2 days. The samples were grounded to fine powder with mortar and pestle. Powder samples (0.1 g each) were heated at 550°C overnight, and 2 ml 67% nitric acid was added. The samples were diluted to a final volume of 25 ml with DDW and were read in ICP, iCAP 6300 (Thermo Fisher Scientific, Waltham, MA, United States) against known standards.

### Statistical Analysis

Statistical analysis was performed using GraphPad Prism 6 software (GraphPad Software, La Jolla, CA, United States). Data were analyzed using one tailed *t*-test; *p* < 0.05 was considered statistically significant.

## Results

We used two complementary approaches to investigate the effect of elevated CK levels on enhanced plant productivity under stress conditions: (A) developing transgenic tobacco plants carrying the *IPT* gene driven by a stress-induced promoter of the *MT* gene; and (B) applying exogenous CKs to elucidate the role of CKs in signal transduction of stress responses and the phosphoproteome of tobacco cells incubated under salinity stress.

### The *MT* Promoter Contains Regulatory Elements Associated With Stress Response

We dissected the *MT* promoter sequence in order to understand the various elements associated with its activation and suppression. Approximately 1.3 Kb genomic sequence upstream of the *Arabidopsis MT* gene was selected as the region of the putative *MT* promoter (Supplementary Figure S2). We identified hypothetical stress-response *cis*-elements in the promoter sequence using PLACE software (Higo et al., 1999)^1^. The identified cis-elements included: eight ARR1 binding elements (5′-GATT-3′) corresponding to CK responses; two MYB recognition sites (5′-AACGG-3′, 5′-GGATA-3′) found earlier in the promoter of dehydration-responsive gene rd22; five MYC recognition sites (5′-CATGTG-3′, 5′-CACATG-3′ and 5′-CANNTG-3′) necessary for expression of ERD1 (early responsive to dehydration) (Simpson et al., 2003); two WRKY (5′-TGAC-3′) elements (Eulgem et al., 2000); four cupper response elements (5′-GTAC-3′) (Quinn et al., 2000); seven ACGT elements required for etiolation-induced expression of ERD1 (early responsive to dehydration); three GT-1 motifs (5′-GAAAAA-3′) that play a role in pathogen and salinity-induced gene expression (Park et al., 2004); and two anaerobic condition elements (5′-GTTTAGCAA-3′ and 5′-AAACAAA-3′) (Mohanty et al., 2005). These potential regulatory elements suggest that the *MT* promoter may respond to various stress conditions. The identified functional cis-elements that could be involved in the regulation of *MT* activation support possible roles in signaling via ABA (the presence of MYB elements), and are also associated with the response to CKs (ARR1). Notably, the cis-element response to different hormones suggests cross-talk between signaling pathways.

### IPT

Expression of Transgenic Plants Carrying MT:IPT Under Various Stress Conditions

**FIGURE 1:**
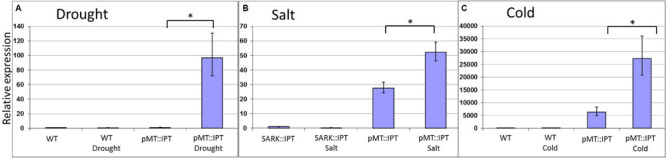
Relative expression of *IPT* in transgenic pMT:IPT and WT in plants under drought **(A)**, salinity **(B)**, and cold **(C)** stress conditions as determined by real-time PCR (RT-PCR). Tobacco plants were grown under optimal conditions for 3 weeks, then they were subjected to cold stress of 4°C for a week. For salinity stress, plants were watered with NaCl solution (100 and then 200 mM) for three more weeks. For the drought-stress treatment, watering of mature tobacco plants was withheld for 3 weeks. Leaf samples were collected simultaneously from all plants (in three independent replicates). Gene expression was determined by RT-PCR. Gene expression in the WT plants under control conditions was used as a reference. Asterisk refers to statistically significant difference between treatments (*p* < 0.05). Values correspond to means ± SE of three independent experiments.

The selected stress-inducible promoter of *Arabidopsis MT* gene was isolated and fused to the *IPT* gene to produce pMT:IPT transgenic tobacco plants (Supplementary Figure S1). The transgenic plants were grown under optimal conditions for 3 weeks (16 h light, 8 h dark). During this period, no significant phenotypic differences were observed between WT and the transgenic tobacco plants. Afterward, the plants were subjected to drought and salinity-stress conditions, each in three independent replications. For the drought stress treatment, watering of mature tobacco plants was withheld for 3 weeks. Plants were subjected to salinity stress by irrigating with 300 mM NaCl solution for 3 weeks. The expression levels of the *IPT* gene in pMT:IPT transgenic plants was significantly higher under both abiotic stress conditions compared to non-stressed conditions (Figure 1), thereby validating the selective regulatory action of the *MT* promoter under these stress conditions.

### pMT:IPT Plants Exhibit Drought Stress Tolerance

**FIGURE 2:**
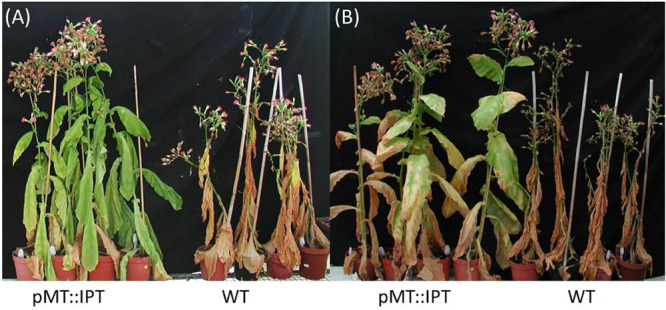
Responses of transgenic pMT:IPT compared to WT plants during drought-stress treatment. Three-month old tobacco plants were subjected to a drought stress of 2 or 3 weeks. **(A)** Withholding watering for 2 weeks. **(B)** Three weeks of withholding watering followed by 1 week of re-watering. Pictures represent one of three independent experiments showing similar results.

Mature transgenic pMT:IPT and WT plants were grown in a greenhouse under optimal conditions (25°C, 16 h light, and 8 h dark) for 3 months, and were then subjected to drought stress. Following 3 weeks of water withholding, WT plants wilted and turned yellow. In contrast, pMT:IPT plants exhibited partial turgor loss, yet, displayed reduced senescence and wilting symptoms (Figure 2). These results suggest a survival advantage for the transgenic CK overproducing plants in the field especially after short drought episodes.

**FIGURE 3:**
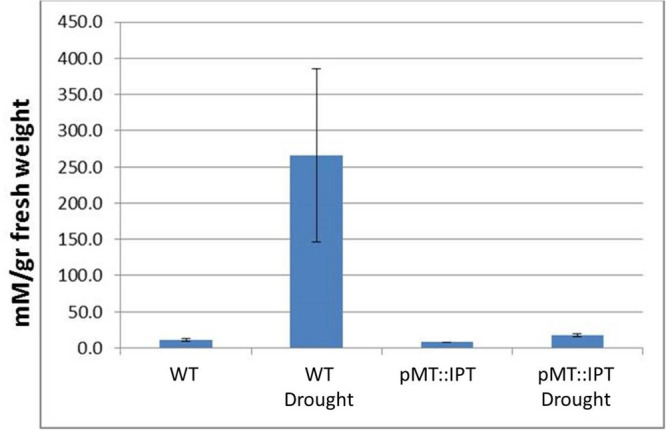
Proline content in pMT:IPT and WT tobacco plants under drought stress. Proline content was determined in leaves taken from mature, 3-month old tobacco plants subjected to drought stress (water withholding for 3 weeks). Proline content was determined by colorimetric assay. Asterisk refers to statistically significant difference between treatments (*p* < 0.05). Values correspond to means ± SE of three independent experiments.

The content of proline, known as an osmoprotectant, was determined in leaves of 3-month old plants. Tobacco plants subjected to drought stress conditions were analyzed for proline levels in three independent experiments. The colorimetric assay results indicated, that proline content increased dramatically, as expected, in WT plants under drought stress conditions probably due to enhanced proline biosynthesis (Bates et al., 1973). A minor increase in proline levels was detected in pMT:IPT plants under stress conditions compared to non-stressed plants (Figure 3), further supporting the stress tolerance of transgenic pMT:IPT plants.

### pMT:IPT Transgenic Exhibit High Tolerance to Salinity Stress Conditions

**FIGURE 4:**
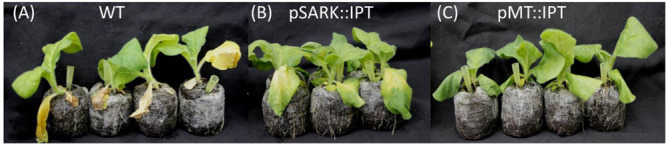
Responses of transgenic compared to WT tobacco plants after salinity stress. Three-week old tobacco plants watered with NaCl solution (100 and then 200 mM) for 3 weeks. **(A)** WT plants, **(B)** pSARK:IPT plants, **(C)** pMT:IPT plants. The photo represents one of three independent experiments showing similar results.

Abiotic stress tolerance of pMT:IPT plants to salinity stress was compared to that of pSARK:IPT and WT plants. Transgenic and WT plants were grown in the temperature- regulated green house for 3 weeks under optimal conditions (16 h light, 8 h dark). The plants were irrigated for three more weeks with salt solution (100 mM NaCl for the first 10 days and 200 mM NaCl for the following 11 days) until yellowing and wilting symptoms appeared in WT plants. Under these conditions, pSARK:IPT transgenic plants displayed mild yellowing, whereas the pMT:IPT plants stayed green (Figure 4) in all the three independent experiments. This observation suggests higher degree of salinity-stress tolerance of the pMT:IPT compared to SARK IPT plants.

**FIGURE 5:**
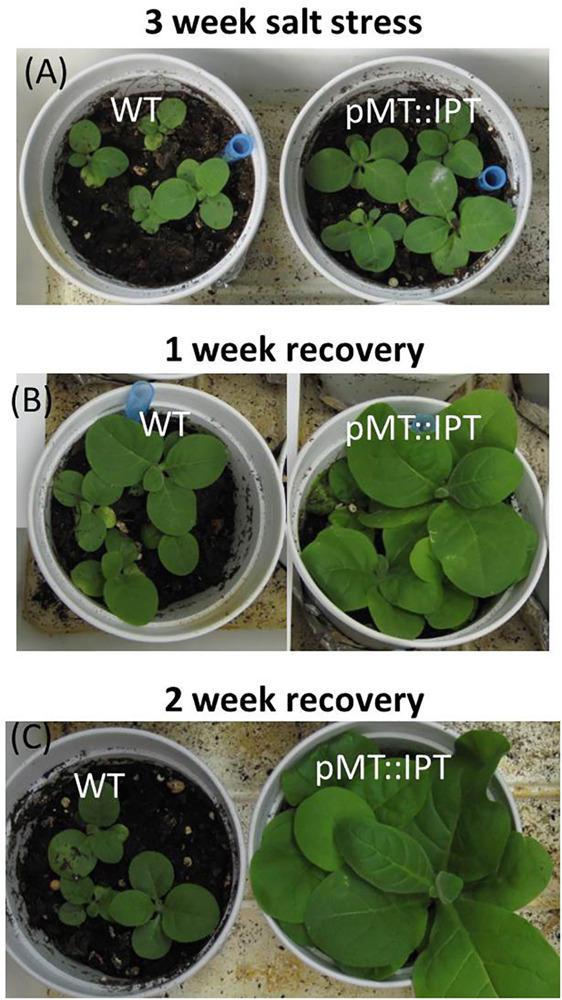
Recovery of pMT:IPT compared to WT tobacco plants after salinity stress. Three week old tobacco plants watered with NaCl solution (100 and then 200 mM) for 3 weeks then transferred to normal watering for two additional weeks. Plants after: **(A)** salinity stress; **(B)** 1 week recovery; **(C)** 2 weeks of recovery. The photo represents one of three independent experiments showing similar results.

In addition, pMT:IPT and WT plants were subjected to salinity stress (100 mM NaCl followed by 200 mM NaCl) for 3 weeks and then irrigated with tap water for recovery. The fast recovery of the transgenic pMT:IPT, as can be seen by the improved growth and biomass development already after 1 week (Figures 5B,C) whereas, WT plants grew much slower and did not seem to recover. These results emphasize the high salinity-stress tolerance of the pMT:IPT tobacco plants.

**FIGURE 6:**
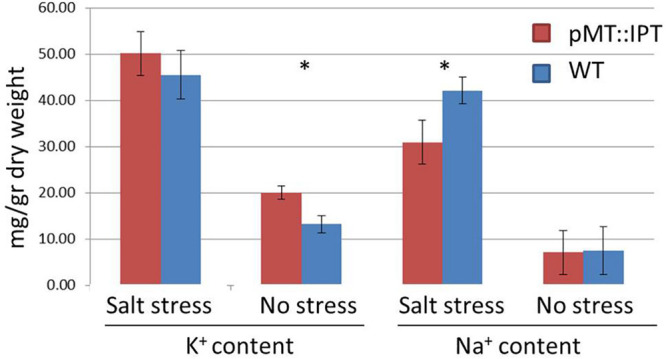
Na^+^ and K^+^ content in pMT:IPT and WT tobacco plants under salinity stress and non-stressed conditions. Three-week old tobacco plants were watered with NaCl solution (100 and then 200 mM) for 3 weeks. Asterisk refers to statistically significant difference between treatments (*p* < 0.05). Values correspond to means ± SE of three independent experiments.

Next, Na^+^ and K^+^ levels were determined under salinity-stress conditions in both WT and pMT:IPT plants as well as under non-stressed conditions. A significant decrease in Na^+^ content was detected in the foliage of pMT:IPT compared to WT plants (Figure 6), whereas no differences were observed between these plants under non-stressed conditions. A slight increase in K^+^ content was found in pMT:IPT plants compared to WT plants under both conditions. Based on the observed reduction in Na^+^ accumulation and the increase in the K^+^/Na^+^ homeostasis, we suggest that pMT:IPT plants maintain plant productivity under salinity stress.

### Expression of Candidate Genes Under Stress Conditions

**FIGURE 7:**
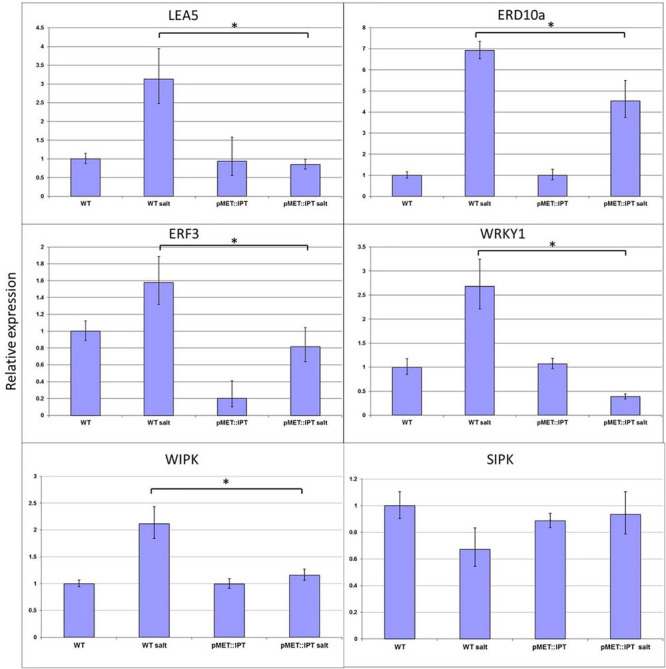
Relative expression of *LEA5*, *ERD10a*, ERF3, *WRKY1*, *WIPK*, and *SIPK* genes in WT and pMT:IPT tobacco plants during salinity stress. Tobacco plants were grown under optimal conditions for 3 weeks and then were watered with NaCl solution (100 and then 200 mM) for three more weeks. Leaf samples were collected simultaneously from WT and pMT:IPT plants (in three independent replicates). Gene expression was determined by real-time PCR. Gene expression in the WT plants under control conditions serves as the reference. Asterisk refers to statistically significant difference between treatments (*p* < 0.05). Values correspond to means ± SE of three independent experiments.

The expression levels of various candidate stress-related genes, in WT and in pMET:IPT plants under normal and salinity-stress conditions were analyzed. The selected candidate genes represent different pathways known to be related to abiotic stress responses: (1) Chaperones: the *NtERD10a* (Early Response to Dehydration) containing DRE/CRT element and belong to Group 2 of the LEA gene family (Kasuga et al., 2004), and *LEA5* that belongs to the same gene family known to maintain the cell membrane integrity under osmotic stress; (2) TFs: The tobacco *WRKY1*, the homolog of *Arabidopsis WRKY33* reported to respond to abiotic stresses in general and to salinity stress in particular (Jiang and Deyholos, 2009), and the *ERF3* (Ethylene responsive factor), an ethylene-related gene, which is known to be upregulated during salinity or drought stresses (Fujimoto et al., 2000; Song and Galbraith, 2006); and (3) Kinases: two MAPK components, *SIPK* and *WIPK* whose expression is known to be upregulated during abiotic stresses (Wu et al., 2007). As expected, all examined genes except *SIPK* were strongly expressed during stress in the WT plants (Figure 7), whereas, their expression levels in the pMT:IPT plants were significantly reduced under the same salinity-stress conditions. *WRKY1* expression was lower in pMT:IPT under stress conditions compared to non-stressed plants. The expression level of *NtERD10a* and *ERF3* genes increased dramatically in WT plants when subjected to salinity stress. However the pMT:IPT plants exhibited lower expression levels under salinity conditions as compared to WT. No significant change in the expression pattern of *SIPK* was observed. These results indicate reversal of the reprograming of the stress responses by CK overproduction as reflected by lowering the expression of *LEA5*, *NtERD10a*, *ERF3*, *WRKY1*, and *WIPK* in pMET:IPT under stress conditions.

### CKs Deactivate Components of the ABA-Dependent and Independent Pathways in Tobacco Cell Suspension Under Salinity Stress

**FIGURE 8:**
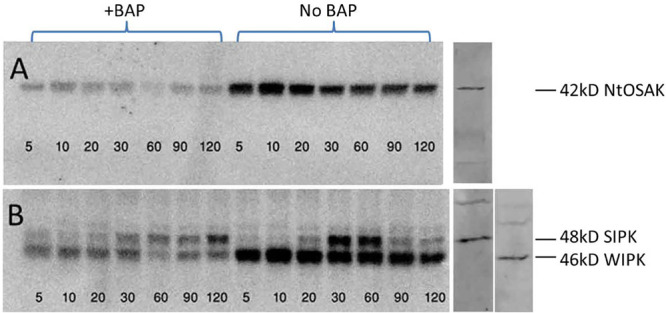
SIPK, WIPK, and NtOSAK kinases activity during salinity stress. Tobacco BY-2 cells were grown for 5 days (at dark, 25°C). The suspension was divided into 250 ml flasks (50 ml suspension in each). After 1 h of pre-treatment incubation, NaCl was added to a final concentration of 50 mM and for the CKs treatments, 6-benzyloaminopurine (BAP), a synthetic CK, was added to final concentration of 10 μM. Samples were collected at indicated times (min). **(A)** In-gel phosphorylation activity of the histone substrate OSAK, and **(B)** phosphorylation of MBP by SIPK and WIPK. Protein ladder 10–180 kDa (Thermo Fisher Scientific, Waltham, MA, United States) was employed for MW estimation as described in “Materials and Methods” section. Kinases were identified by western blot analysis using specific antibodies (right panel) as described in “Materials and Methods” section.

Under conditions of osmotic stress, ABA is generally considered a stress hormone, and expression of stress-responsive genes in plants is primarily regulated by ABA-dependent and -independent pathways. Kinases are known to play a major role in transducing extracellular stimuli into intracellular responses and are rapidly activated following stress both through the ABA dependent and independent pathways. BY-2 tobacco cell suspensions enabled direct addition of the NaCl solution, easier penetration into cells, and faster response as compared to mature intact plant. Kinetic assay of the kinase activity was employed to track rapid changes occurring during signal transduction after adding the external stimuli. To assess the influence of CKs on the phosphorylation of the components of the MAPK kinase cascade (WIPK and SIPK), and on the NtOSAK, cells were exposed to BAP, a synthetic CK, for 1 h and were then subjected to salinity stress for one additional hour. Kinase activity was determined by in-gel kinase assay (Zhang and Klessig, 1997; Wu et al., 2007) using myelin basic protein as a substrate which enabled measuring the phosphorylation activity of WIPK, SIPK, or histone as a substrate for NtOSAK, the tobacco homolog of the SnRK2 protein kinases. Kinases were identified using specific antibodies reacting with protein extracts of BY-2 cell suspension (Seo et al., 2007; Wu et al., 2007). *WIPK* and *SIPK* (members of the MAPK family), are components of the ABA-independent pathway and have been reported to be activated under abiotic stress (Mikolajczyk et al., 2000; Zhang et al., 2000; Seo et al., 2007). Our results indeed confirmed their stress-induced activation, but also demonstrated reduced kinase activity during stress in tobacco cell suspension treated with exogenous CKs (Figure 8). Similar CKs inhibitory effects were demonstrated on the phosphorylation activity of the NtOSAK under salinity stress in the cell suspension incubated with BAP (Figure 8). The in-gel assay showed a clear reduced auto phosphorylation activity of the three identified kinases (WIPK, SIPK, and NtOSAK) as a result of adding BAP to the tobacco cells (Figure 8). These results suggest CK-dependent deactivation of the phosphorylation activity of kinases belonging to both ABA-dependent and -independent pathways acting under abiotic conditions.

### Modifications in the Phosphoproteome of Stress Response by CKs

A range of post-translational modifications (PTMs) have been linked to plant stress responses. Of these modification, perhaps the most studied is protein phosphorylation, which influences development, metabolism, transcription, translation, proteolysis, homeostasis, and signaling (Hu et al., 2015). We analyzed the phosphoproteome of BY-2 tobacco cells under salinity stress to test the post-translational modifications and to further investigate the effect of CKs on the wide response of protein phosphorylation to the stimuli. Approximately 1547 phosphopeptides were identified, collectively containing 1667 non-redundant phosphorylation sites. Of these sites, 83.8% were phosphorylated at serine residue, 13.5% at threonine, and 2.6% at tyrosine. The distribution of phosphor-Ser (pS), phosphor-Thr (pT), and phosphor-Tyr (pY) was consistent with that of *Arabidopsis* (Nakagami et al., 2010). Of the unique phosphopeptides, 1430 were singly phosphorylated, 105 were doubly phosphorylated, and 9 were phosphorylated at three sites. To identify the corresponding proteins of these phosphopeptides, we analyzed the data using MaxQuant versous database combined from *Nicotiana tabacum*, *Arabidopis thaliana, Solanum lycopersicum*, and *Solanum tuberosum*. The fold-change was calculated for each phosphopeptide. The total number of differentially changed phosphoproteins was 61 (>4-fold change, *p* < 0.05). Next, we analyzed their biological function. We were able to predict the identity and function of 26 proteins (Table 1). The remaining proteins were either unidentified proteins (23) or with unknown functions (13).

**TABLE 1:** Identified phosphoproteins of the BY-2 cells under salinity stress^*a*^.

**Protein name**	**Function**	**Biological process**	**Sequence identifier^*b*^**
**De-activated stress-related phosphoproteins are due to CK**			
MAPK16	MAP kinase cascade	Stress signals	gi565404224
PBL	Disease immune response	Stress signals	gi565396124
MKP1	MAP kinase phosphatase	Stress signals	gi37951311
ERS	Suppress ethylene response	Stress signals	gi7652766
eIF4A	RNA helicase	Replication and translation	gi485943
SR	Pre-RNA splicing	Replication and translation	gi440135805
Histone H3	Mitosis and meiosis	Replication and translation	gi218744593
DNA topoisomerase II	dsDNA replication	Replication and translation	gi26984168
Nucleolin	Cell division	Replication and translation	gi21700195
Histone H1	Mitosis and meiosis	Replication and translation	gi90654966
extracellular cationic peroxidase	Response to ROS and stress	Stress response	gi575603
A/N-InvD	Cytoplasmic neutral invertase	Stress response	gi671775156
NF-Yb-10	Nuclear factor Y Transcription Factors Respond to Abiotic Stress	Stress response	gi565392153
Chl-PGK	Chloroplastic Phosphoglycerate kinase – photorespiration	Stress response	gi298541491
AHA	H^+^ ATPase – regulate cytosolic pH under stress	Stress response	gi9789539
GST	Glutathionine S-transferase, oxidative stress responses	Stress response	gi676880
NDPK2	Nucleotide diphosphate kinase 2, reduces accumulation of H_2_O_2_	Stress response	gi565361307
PEPC	Participate in plants responses to limited water supply	Stress response	gi565363147
PBS1-like	Plant immune response	Stress response	gi460387273
**Activated stress-related phosphoproteins are due to CK**			
cycD3-1	Cell divisions	Cell divisions	gi568214824
Histone deacetylase	Histone deacetylase, gene repression	Replication and translation	gi269969916
Ribosomal protein s6	Regulating cellular metabolism and protein synthesis	Replication and translation	gi82623405
Trehalose-6-P Synthase	Regulator of Glucose, Abscisic Acid, and Stress Signaling	Stress response	gi565396921
CPL	Repress stress-response genes	Stress response	gi460401650
MCM	DNA replication	Replication and translation	gi311819860
Centrin	Cell divisions	Cell divisions	gi6358511

*^*a*^BY-2 cells were pre-incubated with 10 μM BAP for 1 h, followed by 30 min of 50 mM NaCl treatment. ^*b*^NCBI GenInfo identifier (gi).*

The identified phosphoproteins were classified by the biological processes of each gene product according to annotations in the NCBI databases. Among the proteins whose activity was reduced due to BAP addition during salinity stress, four are related to stress signal transduction, seven to stress response, and five to replication and translation. Thus, for 11 stress-related phosphoproteins, CKs lead to clearly reduced activation. Among the proteins whose activity was increased due to BAP addition during salinity stress, four are associated with defense of the photosynthetic system and antioxidant activity, two are related to stress responses, three are related to replication and translation, and two are related to cell division (Table 1). These results reinforce the preceding results that CKs reduce the response to stress conditions.

## Discussion

### Stress-Inducible Promoter

Applying exogenous CKs as well as enhanced endogenous CK biosynthesis delay the process of plant aging (Gan and Amasino, 1995; Ha et al., 2012). The development of transgenic plants overexpressing CKs by inducible promoters linked to key genes of CK biosynthesis (such as *IPT*) offer a promising strategy to increase biomass and crop yield by delaying the natural senescence process. While we were studying the delayed aging behavior of the CK-overproducing transgenic tobacco plants (SARK-IPT), we forgot to water the transgenic plants unintentionally and to our surprise, they exhibited dramatic survival under these drought conditions (Rivero et al., 2007). This observation suggested that the phenomenon of CK-induced delayed senescence is also accompanied by an additional advantage of enhanced drought resistance and allowed us to suggest a novel technology for the development of drought-resistant plants (Gepstein et al., 2017). Since then, major crops, including rice, wheat, peanut, cotton etc, have been developed to overexpress CKs under stress conditions (see Hai et al., 2020).

In this study, we designed and developed auto-regulatory CK-overproducing transgenic tobacco plants by expressing the *IPT* gene under the control of a stress-inducible promoter of the *Arabidopsis MT2a* gene. It has been reported that although different signals such as biotic and abiotic environmental stress lead to initiation of stress-induced senescence due to distinct signal transduction pathways, they may share common execution events (Guo and Gan, 2012). We presume that a stress-inducible promoter would have an advantage over age-dependent promoter in the context of conferring synchronous seed set and appropriate harvest timing, which are crucial for best yields and post-harvest storage (Lukac et al., 2012). Choosing the suitable promoter for auto-regulated CKs biosynthesis is critical for normal development of plants prior to the senescence stage (Ma, 2008). Constitutive overexpression of *IPT* under the transcriptional control of CaMV35S promoter showed developmental abnormality probably due to CK supraoptimal levels (Smart et al., 1991; Ma, 2008; Guo and Gan, 2012). pMT:IPT transgenic tobacco plants produced in the present study displayed normal development, but as expected, exhibited enhanced biomass under abiotic stress conditions. Since CK levels are known to decrease under abiotic stress conditions, and optimal CK levels are required for normal plant development, we assume that the *MT* promoter (in contrast to CaMV35S constitutive promoter) is a suitable promoter for driving the expression of *IPT* gene under stress conditions, as it resulted in enhancing CK biosynthesis up to the optimal levels for normal development.

Bioinformatics analysis of the *Arabidopsis MT* promoter suggests the presence of multiple cis-acting elements (Supplementary Figure S2). Among the identified cis-acting elements is the ACTG. Plant genome studies revealed that the sequence motif ACGT is functionally important in a variety of promoters that respond to different stimuli such as light, anaerobiosis, jasmonic acid, and hormones including salicylic acid (SA), ABA, and auxin. This core element is present at different relative positions in multiple copies upstream of the transcription start site (Rushton et al., 2010; Mehrotra et al., 2013).

### Reprograming of Gene Expression in the CKs Overproducing Plants Under Stress Conditions

The role of CKs in the regulation of abiotic stress responses has been elucidated mainly during the last decade (Zwack and Rashotte, 2015; Hai et al., 2020). However, since, surprisingly, plants with exogenous CKs or biotechnological manipulation of CK endogenous levels demonstrated both positive and negative effects on the tolerance to abiotic stresses, generalizations about the effect of manipulating CK levels on overall stress tolerance are currently difficult to make (Zwack and Rashotte, 2015; Hai et al., 2020). Our studies based on employing the SARK or the MT promoter fused to the *IPT* gene clearly demonstrated positive effect on growth and biomass production under abiotic stresses in model plants such as *Arabidopsis* and tobacco as well in major crops such as rice, wheat, cotton, etc. (see Gujjar and Supaibulwatana, 2019; Hai et al., 2020). Similar positive regulation of drought stress by CKs has been reported also for transgenic plants containing the SAG12 promoter fused to the *IPT* gene (Merewitz et al., 2010; Zhang et al., 2010). In apparent contrast to these findings, *Arabidopsis IPT* mutants, which have rather reduced CK levels, are also more drought resistant compared to WT (Nishiyama et al., 2011). Decreased CK levels achieved by overproduction of the CK-degrading enzyme CK oxidase (CKX) in either a constitutive or root-specific manner, also have a positive effect on drought-stress tolerance (Werner et al., 2010; Nishiyama et al., 2011; Mackova et al., 2013). Plants lacking two CK receptors have enhanced tolerance to drought treatment as well as increased sensitivity to ABA (Tran et al., 2007), suggesting that these receptors and presumably also the downstream output of the CK signal negatively impact drought tolerance. Thus, CK-mediated plant tolerance to drought could be achieved through two seemingly contradictory approaches. This is probably due to the choice of promoter and type of tissue modulated for transgene expression, which led to the regulation of different pathways. Our findings, which led us to the present hypothesis of desensitization of environmental cues followed by CK-dependent reprograming of gene expression under stress is aligned with the positive regulation approach of stress tolerance.

Our data indicate that the stress-induced CK overproduction in the pMT-IPT transgenic plants altered the regular pattern of stress-induced growth inhibition to a more moderated mode of inhibition. To elucidate the molecular events associated with CK influence on plant stress responses, we examined some candidate genes known to be involved in different stress-responsive pathways. Expression levels of selected candidate genes reported to be upregulated under stress conditions (Ingram and Bartels, 1996; Kasuga et al., 2004; Hundertmark and Hincha, 2008), displayed opposite results. The general expression pattern of most of the examined stress-related candidate genes in the present study is characterized by lower expression in the transgenic plants as compared to WT plants exposed to identical environmental stress conditions (Figure 7). *LEA5* and *ERD10a* both belong to group 2 of the LEA family. *LEA5* maintains the cell membrane under osmotic stress (Ingram and Bartels, 1996; Hundertmark and Hincha, 2008). *ERD10a* is induced by DREB1 and it is upregulated during stress (Kasuga et al., 2004). It is thought to be a chaperone that defends the cell from aggregation or inactivation of different elements (Kovacs et al., 2008). The expression of these genes was lower in transgenic plants compare to WT under stress conditions. The results of the present study are consistent with our previous experiments describing the transcriptomic analysis of CK-overexpressing *Arabidopsis* plants (Golan et al., 2016). It has been shown that under salinity stress CK triggered transcriptional reprograming that resulted in attenuation of gene clusters related to stress-dependent inhibition of growth and delayed premature plant senescence. In contrast, elevated CK levels led to stress tolerance by retaining the expression of gene clusters associated with plant growth and metabolism whose expression typically decreases under stress conditions (Golan et al., 2016). Since several known pathways related to abiotic stress plant responses are regulated by various plant hormones, it would not be surprising to reveal contradicting results related to expression patterns of specific genes. Gupta et al. showed that genes associated with abiotic environmental stresses were differentially induced by CKs which is not consistent with previous reports (Gupta et al., 2013). The peroxidases, several of which were found to be repressed while others were found to be induced by CKs indicate a complex role for CKs in the regulation of abiotic stress related responses (Passardi et al., 2005).

### Signal Transduction of Environmental Stress Stimuli

Plants as non-mobile organisms constantly integrate varying environmental signals to flexibly adapt their growth and development. Local fluctuations in water, sudden changes in other abiotic factors, and stresses can trigger changes in the growth of plant organs. The molecular backbones of signaling cascades of individual hormones have been established; however, it is well accepted now that multiple mutually interconnected hormonal signaling cascades act as essential endogenous translators of these exogenous signals in the adaptive responses of plants (Verma et al., 2016). Hormonal pathways connected via multiple levels of interactions form powerful regulatory networks that sensitively react to changes in the environment and drive the relevant adaptive responses (Verma et al., 2016). Upon sensing environmental stresses, plants sacrifice growth and activate protective stress responses such as the avoidance syndrome. These include transcriptional changes, phytohormone synthesis and degradation, and finally the direct/indirect molecular defense mechanisms that are translated into developmental and morphological changes. Kinase (including MAPK) cascade is a conserved signaling pathway involved in modulating many cellular responses in all eukaryotes and plays a major role in transducing the extracellular stimuli into intracellular responses. Accumulated evidence indicates that in plants, MAPKs also regulate TFs at both transcription and protein activity levels. The SA-induced protein kinase (SIPK) of tobacco, which is a MAPK, is activated by various biotic and abiotic treatments and has been found to phosphorylate the transcription factor WRKY1 (Menke et al., 2005). In Addition, the involvement of *SIPK*/*WIPK* has been demonstrated in the cell death signaling pathway mediated by phosphorylation of WRKY1 in tobacco plants (Ogata et al., 2015). In general, plant hormones affect similar processes but their signaling pathways act non-redundantly and their signals are integrated at the gene network level (Jaillais and Chory, 2010). Since the interplay between hormone levels and signaling networks plays a crucial role in the trade-off between plant growth and stress adaptation, the antagonistic relationship between ABA and CK signaling should be considered in the context of stress responses. Recent studies provides several lines of evidence showing crosstalk between ABA and CK signaling under abiotic stresses (Huang et al., 2018). Our results emphasizing the inhibitory effect of CKs on the phosphorylation of NtOASK, a homolog of the SNF1-related protein kinases (SnRK2) the key kinase of the ABA signaling pathway. These results are consistent with the study demonstrating that SnRK2 directly interacts with and phosphorylates type-A response regulator 5 (ARR5), a negative regulator of CK signaling (Huang et al., 2018). The results of the kinetic experiments of kinase activities as viewed in the in-gel kinase activity system, suggest a dramatic negative influence of CKs on the rate of phosphorylation of several components of the kinase cascade that are known to be activated under stress conditions. Similar in-gel kinase activity was performed in *Nicotiana attenuata* and the identity of each of the kinases was validated by specific antibodies in a Western blot analysis (Wu et al., 2007). Activity of NtOASK, SIPK, and WIPK was dramatically reduced by adding exogenous CKs under salinity stress (Figure 8), which may indicate suppression of at least several components of the signal transduction of environmental stress cues. In addition, our previous gene expression study suggested that the major regulatory ABA signaling component *SnRK2.3* was among the genes whose expression increased under salinity stress and was attenuated by CK (Golan et al., 2016). The present results confirm previous studies that demonstrated that NtOSAK (*Nicotiana tabacum* osmotic stress-activated protein kinase), a member of the SnRK2 subfamily, is being activated rapidly in response to hyperosmotic stress (Burza et al., 2006). The elevated phosphorylation activity of NtOSAK due to salinity- stress conditions was reduced upon the addition of CKs (Figure 8). Taken together, not only the NtOSAK phosphorylase activity is dramatically reduced by CKs, the transcription of its *Arabidopsis* homolog gene *SnRK2* is reduced as well (Golan et al., 2016).

**FIGURE 9:**
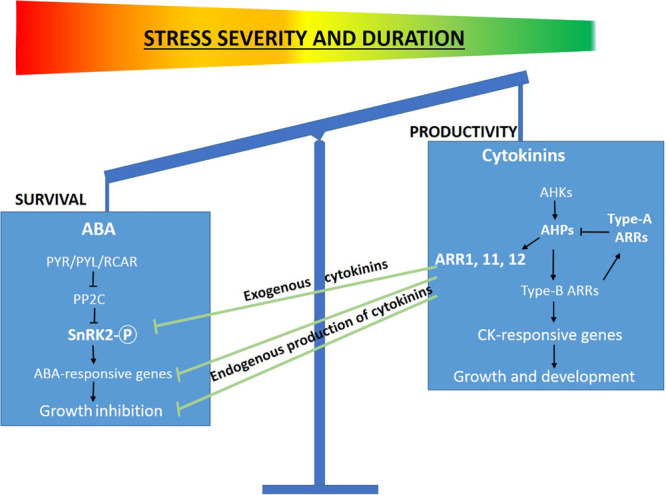
The balance between stress tolerance and productivity modes is reflected by the antagonistic action of ABA and CK signaling. Schematic representation of the working model demonstrating the regulatory role of CKs in promoting productivity under abiotic stress by inactivating SnRK2 the key component of ABA signaling pathway. Under stress conditions, CKs inactivate SnRK2 and inhibit ABA signaling pathway and the downstream upregulation of stress related genes and growth promotion via CK-responsive genes. The model suggests a molecular mechanism underlying the behavior of CK over-expressing transgenic plants under mild abiotic stress conditions for short periods. This model has been partially adapted from Claeys and Inze (2013).

We hypothesize that the antagonistic inputs of ABA and CKs balance the survival mode of growth inhibitory responses and allow the productivity mode required for biomass production (Figure 9). Several molecular components of the ABA and CK signaling pathways have been shown to mediate drought stress response and suggest possible regulatory mechanisms responsible for coordinating growth and stress responses. A suggested model depicts how ABA and CKs antagonistically regulate growth processes and environmentally stress responses (Claeys and Inze, 2013). Our assumption that CKs reduce the stress-induced growth and productivity inhibition by desensitization of environmental cues is partially based on the reduced phosphorylation activity of MAPK signaling components (WIPK, SIPK) and of the major component of the core ABA signaling pathway (SnRK2).

### Phosphoproteome of the CK-Overproducing Plants Under Abiotic Stress

Protein phosphorylation is a widely used mechanism of post-translational modification that controls the protein activity, stability, turnover, subcellular localization, and interaction with partner proteins including signal transduction of external stimuli. Plant growth adjustment during water deficit is a crucial adaptive response. The rapid fine-tuned control achieved at the post-translational level is believed to be of considerable importance for regulating early changes in plant growth reprogramming. ABA-dependent and -independent pathways are responsible for activating many of plant responses to abiotic stresses including post- translational modifications (Zhu, 2016). In the core ABA signaling pathway, ABA binding to the PYL family of receptors causes the receptor to interact with and inhibit the PP2Cs, leading to release of suppression and activation of the SnRK2s. SnRK2 phosphorylates at least several dozens of downstream effector proteins of ABA action. These phosphorylated effector proteins function in regulating gene expression at various steps, including epigenetic and transcription factor regulation, RNA splicing, and mRNA cleavage or translational repression by miRNAs (Zhu, 2016). Aiming at a better understanding of the CKs influence on the responses to salinity stress in tobacco cells, we carried out a survey of protein phosphorylation events following the addition of exogenous CKs. Adding CKs during salinity stress caused extensive changes in phosphorylation status in critical regulators that are either directly or indirectly involved in plant growth and development (Table 1). Among the identified proteins whose activity was reduced due to BAP addition during salinity stress, were those related to major pathways of stress signal transduction, stress responses, and replication and translation. Among the proteins whose phosphorylation status was increased due to BAP addition during salinity stress, were those associated with defense of the photosynthetic system, antioxidant activity, stress responses, replication and translation, and cell division. The systematic phosphoproteomic study related to plant defense mechanisms under abiotic stress could complement the molecular genetic studies and provide novel insights into mechanisms related to post-translational modifications. Our study indicates that alterations in protein phosphorylation may be the result of the elevation in CKs levels and support our notion related to CK role in sustaining growth under abiotic stress as demonstrated in our present and previous study at the level of gene transcription (Golan et al., 2016).

### Desensitization of Stress Cues Prevents the Avoidance Syndrome Under Stress Conditions

Plants have evolved the stress avoidance strategy to cope with harsh environmental conditions and to avoid fatal damage of plants. Avoiding extinction is driving the evolution of genes that control growth in threatening stress environments. The stress avoidance strategy is translated in annual plants into reduced vegetative growth, early flowering, accelerated senescence, and fast seed set. The obvious advantage is the survival of plants under environmental stress but it is also accompanied by an enormous reduction in plant productivity. Growth inhibition as a dominant response feature results from being exposed to stress due to the genetic programs that have been adjusted to the external circumstances and are geared toward a survival rather than productivity. While altering the expression of regulators of drought responses has often succeeded in enhancing drought tolerance, at least under laboratory conditions, this usually comes at the cost of growth inhibition, resulting in a significant yield penalty (Yang et al., 2010). Similarly, breeding for enhanced water use efficiency leads to impaired plant productivity (Blum, 2009). Hormones mediate the trade-off between plant growth and stress tolerance throughout the life of the plant (Bohnert et al., 1995). Of special interest is the balance between maintained growth on one hand and ensured survival on the other hand. However, most of the breeding programs and genetic engineering have not succeeded to genetically remove these stress responses because they are polygenic and redundantly programmed (Claeys and Inze, 2013; Maggio et al., 2018). Understanding the molecular basis of these trade-offs would provide novel breeding strategies to optimize crop yield (Claeys and Inze, 2013; Huang et al., 2018). Despite these shortcomings, evidence of drought-tolerant crops are being accumulated reviewed in Huang et al. (2018).

Our results demonstrated the striking phenomenon that CKs allow plants to sustain growth even under unfavorable stress conditions. CKs reprogram plants from the survival mode which is fundamentally based on deactivation of the growth mode to a productive mode. The CK-induced inactivation of the kinase cascade components of the stress signal transduction (Figure 8) might explain the prevention of the expression of stress-related genes (Figure 7) and possibly the reduction in the level of the stress-induced phosphoproteomics (Table 1). Our study suggests that CKs cause desensitization of the environmental cues and as a result prevent the activation of the known avoidance mechanisms and consequently allow sustainable growth and productivity under stress conditions. Plants including high-yield crops react over-cautiously to stress conditions and as a result over-reduce growth to be able to survive stresses for a period of time much longer than a cropping season (Maggio et al., 2018). We hypothesize that we improved yield under stress conditions by removing the premature alarms (stress stimuli) that trigger growth reduction and other unproductive responses. Our results demonstrate that under abiotic stress, CKs antagonize ABA signaling pathway by inactivating NtOASK, a family member of the SnRK2, a key component of the ABA signaling pathway and as a result prevent the ABA-dependent growth arrest (Figures 8, 9). However, under the same conditions, CKs activate the known CK signaling pathway and promote plant growth and productivity. These results are consistent with our *Arabidopsis* transgenic plants demonstrating growth maintenance even under stress conditions (Golan et al., 2016). This may be true for most crops grown under mild stress conditions for a short cropping season (Figure 9). Although our experiments were performed not under mild stress, they exhibit this phenomenon probably due to the short period experimentation. However, this manipulation will not solve the problem of growing crops under severe stresses for long periods where the avoidance is required for survival (Claeys and Inze, 2013; Maggio et al., 2018). Our present study performed with tobacco plants supports and supplements our previous conclusions in a study carried out in *Arabidopsis* (Golan et al., 2016), where we showed that CK-dependent desensitization of the stress environmental stimuli might reduce the over sensitivity of plants and allow plants to avoid the evolutionary based avoidance syndrome.

## Conclusion

We have shown that cytokinins-overexpressing plants regulated by senescence or stress-specific promoter, maintain growth and productivity under abiotic stresses due to desensitization of stress environmental stimuli. We hypothesize that CKs allow sustainable plant growth under unfavorable environmental conditions by deactivating key kinases belonging to the signal transduction of the environmental cues and downstream processes leading to prevention of gene expression related to premature senescence and growth inhibition under stress conditions. Manipulated plants seem to avoid the evolutionary-based avoidance plant strategy and change their survival mode of growth inhibition into productivity mode under stress conditions. This approach, based on the autoregulation of CK biosynthesis, may serve as a generic technology for developing various crops for improved productivity and yield under mild environmental stresses.

### Nucleotide Accession Numbers

Nucleotide sequence of MET:IPT_NOS promoter was submitted to the GenBank database under accession number MN862698.

## Data Availability Statement

The mass spectrometry proteomics data have been deposited to the ProteomeXchange Consortium via the PRIDE partner repository with the dataset identifier PXD019418.

## Author Contributions

All authors contributed extensively to the work presented in this paper. AA performed most of the experiments. YeG collected the data and did the bioinformatics analysis. NS established the system of the BY-2 suspension cells. YS carried out promoter isolation and prepared the construct. YaG performed the salinity stress experiments. YD-P assisted with the plant growth experiments and writing the manuscript. SG provided direction and guidance for this project and did the critical revision of the article.

## Conflict of Interest

The authors declare that the research was conducted in the absence of any commercial or financial relationships that could be construed as a potential conflict of interest.

## Footnotes

1 http://www.dna.affrc.go.jp/PLACE

## Supplementary Material

FIGURE S1 Nucleotide sequence of the metallothionein (Metallo) promoter-IPT-NOS terminator construct. MET::IPT NOS 2334 bp: The metallothionein promoter nucleotides 1–1300 (blue letters); the IPT coding sequence nucleotides 1301–2100 (black letters); and the NOS terminator nucleotide 2101–2334 (red letters)(Gepstein, 2013). GenBank accession number MN862698.

FIGURE S2 Nucleotide sequence of metallothionein (MT) promoter. Highlight regions correspond to (1) MYC recognition sites (green); (2) ARR1 binding elements (yellow); (3) cupper response elements (double underlined); (4) WRKY elements (blue); (5) ACGT elements (red); (6) GT-1 motifs (grey); (7) MYB recognition sites (violet); and (8) anaerobic condition elements (teal).

TABLE S1 Real-time PCR primers used for quantification of stress related gene expression.
