# Supplementary figures and images for: From Survival to Productivity Mode: Cytokinins Allow Avoiding the Avoidance Strategy Under Stress Conditions
Source: Front Plant Sci. 2020 Jul 2;11:879. doi: 10.3389/fpls.2020.00879 (PMC7343901; doi:10.3389/fpls.2020.00879)

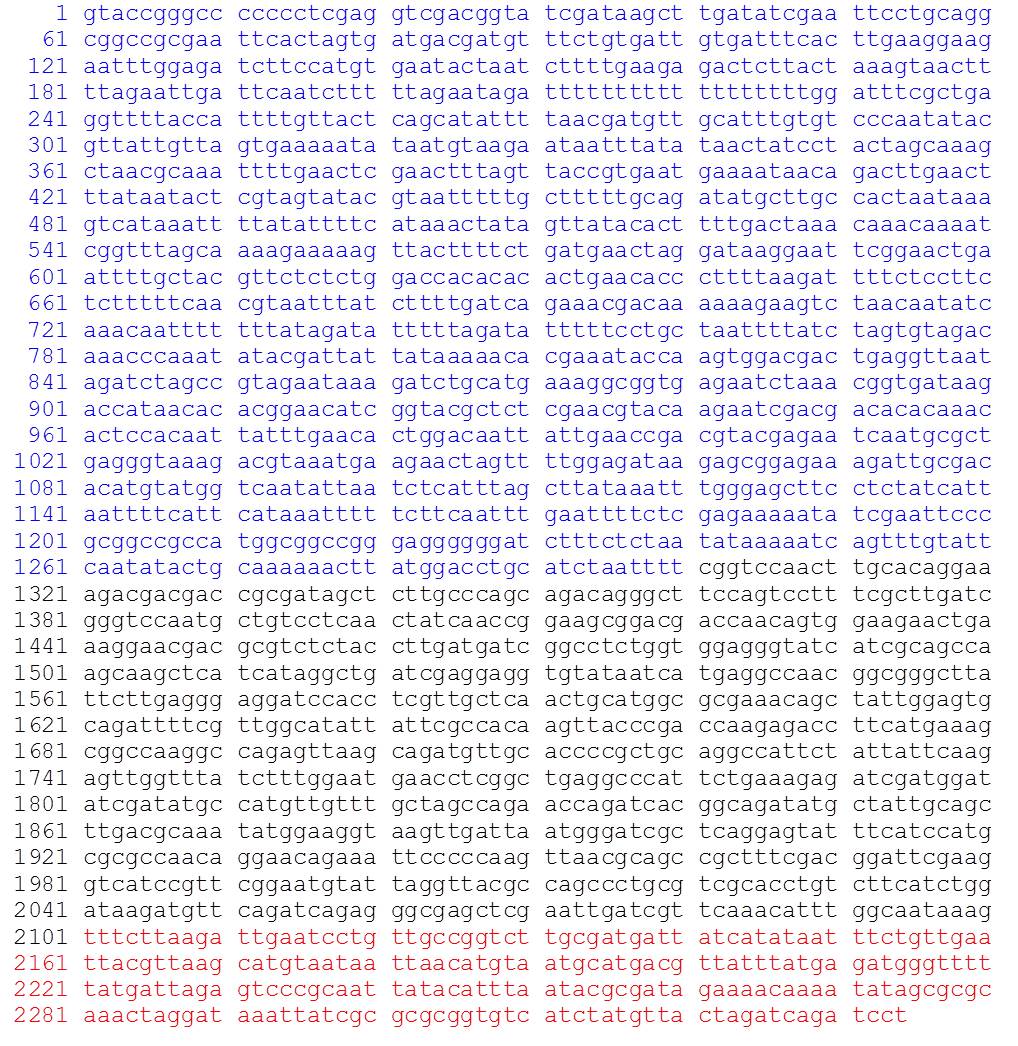

Supplement: FIGURE S1 — Nucleotide sequence of the metallothionein (Metallo) promoter-IPT-NOS terminator construct. MET::IPT NOS 2334 bp: The metallothionein promoter nucleotides 1–1300 (blue letters); the IPT coding sequence nucleotides 1301–2100 (black letters); and the NOS terminator nucleotide 2101–2334 (red letters)(Gepstein, 2013). GenBank accession number MN862698. [file Image_1.JPEG]

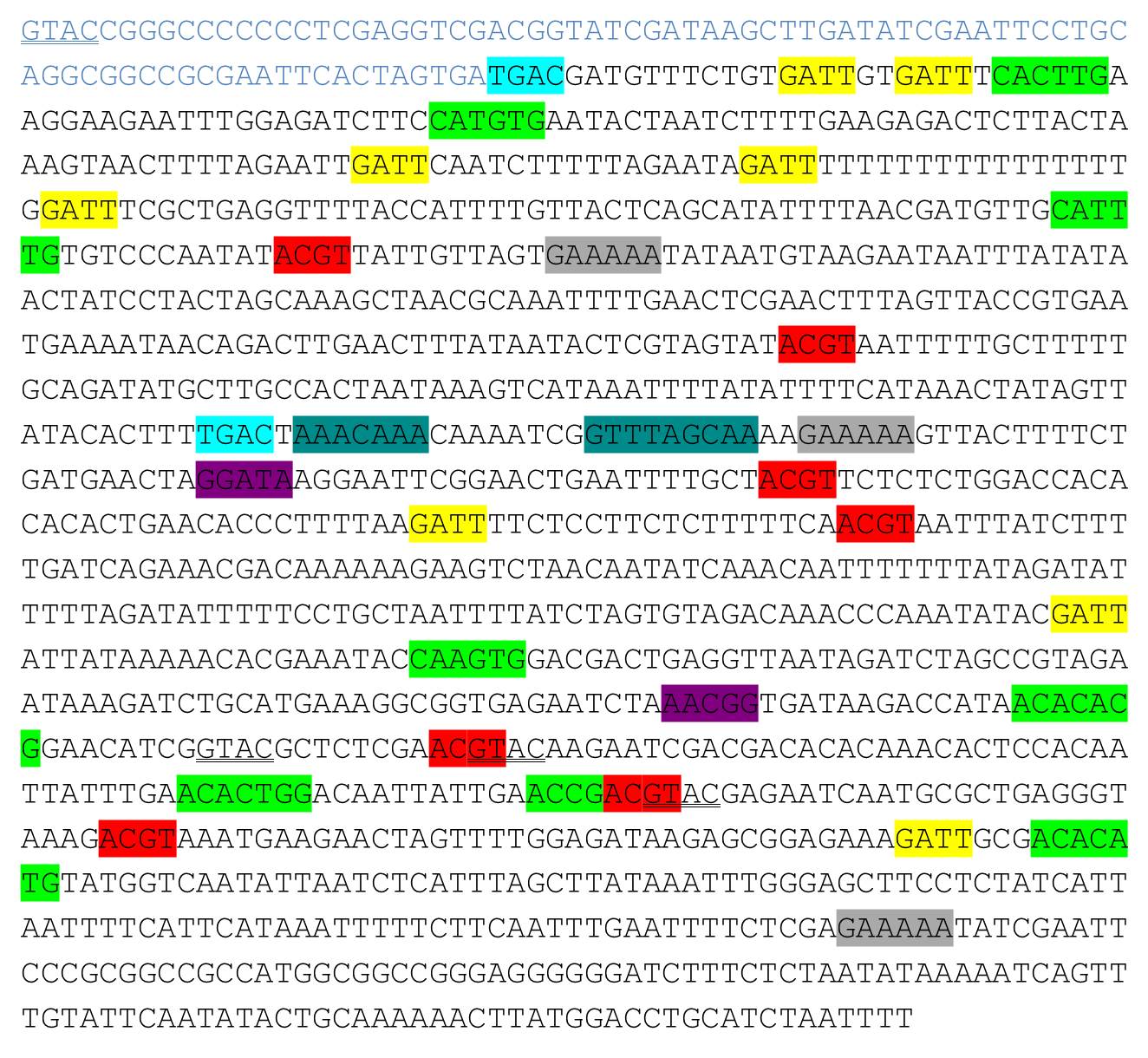

Supplement: FIGURE S2 — Nucleotide sequence of metallothionein (MT) promoter. Highlight regions correspond to (1) MYC recognition sites (green); (2) ARR1 binding elements (yellow); (3) cupper response elements (double underlined); (4) WRKY elements (blue); (5) ACGT elements (red); (6) GT-1 motifs (grey); (7) MYB recognition sites (violet); and (8) anaerobic condition elements (teal). [file Image_2.JPEG]
