# Supplementary material for: From Survival to Productivity Mode: Cytokinins Allow Avoiding the Avoidance Strategy Under Stress Conditions
Source: Front Plant Sci. 2020 Jul 2;11:879. doi: 10.3389/fpls.2020.00879 (PMC7343901; doi:10.3389/fpls.2020.00879)
Supplement: TABLE S1 — Real-time PCR primers used for quantification of stress related gene expression. [file Table_1.pdf]

Table 1S. Real-time PCR primers used for quantification of stress related gene expression.

**Locus**	**Gene / function**	**Forward primer (5'-3')**	**Tm (^{0}C)**	**Reverse primer (5'-3')**	**Tm (^{0}C)**
WIPK	Wound induced protein kinase	CCAAGTATCGTCCTCCTATTATG	52.2	TCACGGAGAGTCCTCTTAGC	55.7
SIPK	Senescence associated induced protein kinase/ salicylic acid-activated MAP kinase	CCACGGTGGCAGGTTCATTC	58.9	CAGAACAAACGATGCCGTAAGC	56.8
NtERF3[1]	ethylene response factor	AGGAATTGATCTTGATCTTAAC	55.3	ACAAAATTCAACCATTAGTCTC	56.3
NtWRKY1[2]	Tobacco probable WRKY transcription factor 26	GATGTTACAGAGCTCTGGAAATTC	61.9	TCGGCTTGATATTATTCATGGGC	67.4
NtERD10a	Early Response to Dehydration	TGAGAAGAAGGGAATTATGGACAAG	54.4	CGCAGCAGATTTTCTAGTGGTG	52.7
18S rRNA	18S ribosomal RNA (AJ236016)	^{1599}AGTCATCAGCTCGCGTTGAC	60.0	^{1665}TCAATCGGTAGGAGCGACG	60.7
CAT1[3]	SR1 salicylic acid binding catalase	CTGAAGCAAAAGAAAGGCAACTAA	54.2	GAATGGAGAATTGAAGGCACTTG	54.4
APX[3]	Ascorbate peroxidase	TCAGTTGAAAAGTGCGAGAGAGG	57.0	GAACCAAAATAGGATGACAAAACGT	54.1
LEA5[4]	late embryogenis abundant protein 5	CGTCGTTGATACTGTTTCTTCCTTT	65.1	GGCACACTAGCTGATGATGCA	66.3

[1] NtERF3- *Nicotiana tabacum* ethylene response factor (Ogata et al., 2012).

[2] NtWRKY1 is a *N. tabacum* homologue of Arabidopsis WRKY33, a transcription factor associated with the response to abiotic stresses (Jiang and Deyholos, 2009).

[3] CAT1 and APX are antioxidant genes (Xing et al., 2008).

[4] LEA5 is known to maintain cell's membrane under osmotic stress (Ingram and Bartels, 1996; Hundertmark and Hincha, 2008)
